# Follow up rates and patient interest in clinical care after mild traumatic brain injury presenting to a level 1 trauma center: a TRACK-TBI prospective cohort study

**DOI:** 10.3389/fneur.2025.1558204

**Published:** 2025-04-02

**Authors:** Shawn R. Eagle, Jason Barber, Nancy Temkin, Michael A. McCrea, Joseph T. Giacino, David O. Okonkwo, Debbie Madhok, John K. Yue, Jennifer M. Zerbato, Geoffrey T. Manley, Lindsay D. Nelson, The TRACK-TBI Investigators, C. Dirk Keene, Vijay Krishnamoorthy, Christine Mac Donald, Randall Merchant, Pratik Mukherjee, Laura B. Ngwenya, Ava Puccio, Claudia Robertson, Richard B Rodgers, Sabrina R. Taylor, Ross Zafonte

**Affiliations:** ^1^Department of Neurological Surgery, University of Pittsburgh, Pittsburgh, PA, United States; ^2^Department of Neurological Surgery, University of Washington, Seattle, WA, United States; ^3^Medical College of Wisconsin, Milwaukee, WI, United States; ^4^Department of Physical Medicine and Rehabilitation, Harvard University, Cambridge, MA, United States; ^5^Department of Neurological Surgery, University of California, San Francisco, San Francisco, CA, United States

**Keywords:** mTBI, emergency department, clinical follow up, concussion, blood biomarkers, GFAP

## Abstract

**Study objective:**

To evaluate the rates of clinical follow-up and patient interest in clinical follow-up within the first year of traumatic brain injury (TBI) with presenting Glasgow Coma Scale (GCS) score between 13 and 15.

**Methods:**

This is a secondary analysis of a prospective cohort study which enrolled patients with TBI first evaluated at a 1 of 23 level 1 trauma centers (*n* = 1,916). At 2 weeks and 3 months, the participants were asked “have you seen any healthcare provider for your TBI?” and “if so, did it help?.” Participants also completed the Rivermead Post-Concussion Questionnaire (RPQ), Quality of Life after Brain Injury- Overall Scale (QOLIBRI-OS), and Glasgow Outcome Scale Extended for TBI (GOSE-TBI) at 2 weeks, 3-, 6-, and 12-months. Persistent symptoms were defined as 3+ symptoms worse than pre-injury levels. QOLIBRI-OS≤51 was defined as lower quality of life. GOSE<8 was defined as incomplete recovery.

**Results:**

By 2 weeks, 43% of participants had followed up with a clinical provider; cumulative follow-up within the first year was 63%. Overall, 61% of participants interested in clinical follow-up care reported receiving clinical follow-up care. Participants who received follow-up care reported that it helped at an 86% rate. Of those not interested in follow-up care, 42% reported receiving clinical follow-up care and 86% of those receiving care reported that it helped. Approximately 44% of participants who reported “I did not think I need follow-up” at 2 weeks had incomplete recovery (GOSE<8), 40% had persistent symptoms, and 19% had lower quality of life at 12-months post-injury.

**Conclusion:**

Participants not interested in follow-up care had high rates of poor functional recovery, persistent symptoms and lower quality-of-life at 12 months following traumatic brain injury with GCS 13–15. Education and provider emphasis on the importance of clinical follow-up after hospital discharge with TBI need to be enhanced. Prioritizing timely clinical follow-up for adult patients with TBI with GCS 13 to 15 is critical for improving rates of long-term recovery in this population.

## Introduction

Traumatic brain injury (TBI) with a presenting Glasgow Coma Scale (GCS) score between 13 and 15 represents 4 in 5 adult patients evaluated for TBI at United States emergency departments (ED) annually (1). Although this GCS score range has historically been classified as “mild,” over 50% of these patients have functional limitations at 1 year post-injury (2). Even in adult patients with a GCS of 15 and negative head computed tomography (CT) scan, 56% have functional limitations at 6-months post-injury (3, 4). One potential reason for these negative prognoses is a lack of standardized care protocols for patients after discharge from the hospital. For athletes with sport-related concussion (SRC), a head injury disproportionally characterized by GCS of 15 and negative head CT scan (5), consensus guidelines advocate for active rehabilitation strategies following 48 h of relative rest (6, 7). This approach is supported by strong evidence that earlier and more active therapies reduce symptoms and recovery time faster than strict rest (7, 8). Consensus guidelines for post-injury management of adults with “mild” TBI first evaluated at a hospital ED do not currently exist. As a result, patients in this population often do not follow up with another medical provider after ED discharge. Seabury et al. (9) reported that only 44% of mild TBI patients followed up with a medical practitioner by 3 months post-injury after discharge from a hospital. The authors also reported that the provision of educational material about mild TBI varied significantly across 11 enrolling hospitals (19–72% of patients) (9). Improving systems of care for patients with TBI after hospital evaluation has become a national healthcare priority, as the National Academies of Science, Engineering and Medicine (NASEM) have convened numerous committees of subject matter expert and stakeholders to address this problem (10, 11). Identification of certain populations for which follow-up care may be especially beneficial could help facilitate effective transitional care from hospital discharge to a secondary level of care.

One such TBI subpopulation are those who present with no objective clinical findings based upon current emergency department standard of care (i.e., negative CT scan, GCS of 15), which represent a majority of adults with mild TBI (12). However, 27% of adults with mild TBI and negative CT scan have positive magnetic resonance imaging (MRI) findings (13), which is considered a more sensitive neuroimaging tool. Conducting MRIs during ED visits is not feasible, due to time and availability constraints. The advent of blood biomarkers to facilitate clinical decision making for patients with TBI represent a critically important time-savings with potentially higher sensitivity than a CT scan (14). In April 2024, the U.S. Food and Drug Administration (FDA) approved the whole blood TBI test of Glial Fibrillary Acidic Protein (GFAP) and Ubiquitin c-Terminal Hydrolase-L1 (UCH-L1) to assist in determining the need for a head CT. Assessment of these blood biomarkers could give another time-expedient, objective assessment for ED providers to communicate the importance of clinical follow-up in patients without a positive head CT scan (13), as many of these patients experience long-term difficulties following mild TBI (3, 4, 15, 16). No study to date has assessed if acute blood biomarker results influence clinical follow up rates, especially in a CT-negative population.

The purpose of this study was to evaluate the rates of clinical follow-up and patient interest in clinical follow-up following discharge from the hospital within the first year of TBI with presenting GCS 13 to 15 and its associated predictors (i.e., demographics, medical history, injury characteristics). The secondary purpose was to understand the relationship between day of injury GFAP and UCH-L1 with rates of follow-up and patient interest in follow-up after TBI.

## Methods

This is a secondary analysis of a prospective cohort study of participants enrolled in the Transforming Research and Clinical Knowledge in Traumatic Brain Injury (TRACK-TBI) study (2013–2019). TRACK-TBI is a prospective, longitudinal, observational study of patients with TBI who presented to the emergency department of 18 level 1 trauma centers in the United States (*n* = 2,697).

### Procedures

Participants or their legally authorized representatives provided written informed consent to participate after being approached by a member of the research team in the ED. Enrolled patients provided blood samples within 24 h of injury. Participants were included in the study if presenting to the ED within 24 h of head injury and had a CT scan ordered. Exclusion criteria included pregnancy, incarceration, nonsurvivable physical trauma, debilitating mental health disorders or neurological disease, magnetic resonance imaging contraindications. For the purposes of the present analysis, we excluded participants <17 years of age (*n* = 145), those with a GCS < 13 (*n* = 552), those who had died by 2 weeks (*n* = 14), and those who had not been discharged from the hospital by 2 weeks (*n* = 70). For sensitivity analyses, any participants with GCS < 15 were excluded (*n* = 944).

Participants completed a standardized outcome assessment battery at 2 weeks and 3 months, including questions about demographic information (i.e., age, sex, race, ethnicity, years of education, insurance type, highest level of care, arrival GCS, loss of consciousness, post-traumatic amnesia, initial CT status, TBI history, psychiatric history, migraine history) and clinical follow-up since their most recent research visit. Participants were asked whether they had been provided educational materials about their injury, whether they had been provided with follow-up care contact information, and whether the hospital system had called to follow-up with the patient. At 2 weeks and 3 months, the participants were asked “have you seen any healthcare provider for your TBI?” and “if so, did it help?.” Participants were also asked if they had received any inpatient or outpatient rehabilitation for their TBI since the injury. At 6- and 12-months, participants were asked the same questions regarding the past 3- and 6-months, respectively (i.e., not since injury but the last research visit). Participants were also asked whether they were interested in follow-up care with the following options: (1) “yes, but I do not have sufficient insurance coverage,” (2) “yes, but insurance coverage was denied,” (3) “yes, but could not arrange transportation,” (4) “yes, but worried about the physical, emotional or personal consequences,” (5) “yes, but worried about the burden it would place on others close to me,” (6) “yes, but treatment options have not been arranged,” (7) “yes, but not given any information or referral,” (8) “no, because I do not think I needed it,” (9) “no, because I believe I can manage my problems on my own,” or (10) “no, because I was dissatisfied with the treatment I received at the hospital.” Participants also completed the Rivermead Post-Concussion Questionnaire (RPQ), Quality of Life after Brain Injury- Overall Scale (QOLIBRI-OS), and Glasgow Outcome Scale Extended for TBI (GOSE-TBI) at 2 weeks.

### Blood biomarkers

Blood samples were collected within 24 h of injury. Samples were processed and stored according to the Traumatic Brain Injury Common Data Elements Biospecimens and Biomarkers Working Group consensus recommendations for plasma and serum preparation (17). The first batch of plasma GFAP concentrations was measured using the prototype point-of-care i-STAT Alinity System (Abbott Laboratories, Abbott Park, IL, USA). The second batch of plasma GFAP concentrations were measured on the prototype core lab ARCHITECT platform (Abbott Laboratories, Abbott Park, IL, USA) for faster throughput. Because the two assays were highly correlated, ARCHITECT values were converted to iSTAT equivalents by use of two previously derived equations prior to analysis (18).

### Statistical analysis

For categorical variables, follow-up rates were provided by 2-weeks, 3-months, 6-months and 1-year based upon demographic characteristics listed above. Follow-up rates were also reported for clinically relevant subgroups, including those who had received multiple hospital contacts/information, those with a day-of-injury GFAP≥100 pg./mL, negative arrival CT scan and GFAP≥35 pg./mL, RPQ total score ≥ 14 at 2 week research visit, GOSE-TBI < 8 at 2 week research visit, and QOLIBRI-OS<51 at 2 week research visit. The GFAP cutoff levels were determined based upon the indicated plasma concentration cutoff for needing a head CT scan (≥35 pg./mL) and the likelihood that a patient has suffered a TBI based upon median values reported in prior research (≥100 pg./mL) (13). The cutoff for RPQ was chosen based upon prior research demonstrating scores over the cutoff at 2 weeks were predictive of worse long-term outcomes. Sensitivity analyses were conducted including only participants with GCS = 15 at arrival and negative head CT scan, including reporting clinical follow-up rates by 2-weeks, 3-months, 6-months and 1-year. Follow-up rates were also reported for clinically relevant subgroups within the sensitivity analysis subset, including those who had received multiple hospital contacts/information, those with a day-of-injury GFAP≥100 pg./mL, negative arrival CT scan and GFAP≥35 pg./mL, RPQ total score ≥ 14 at 2 week research visit, GOSE-TBI < 8 at 2 week research visit, and QOLIBRI-OS<51 at 2 week research visit. Amount of missing data are reported for all outcomes. Because the statistics are primarily descriptive, all cases were included in analyses regardless of missing data. No adjustments were made for multiple comparisons. Statistical significance was set to *p* < 0.05.

## Results

### Descriptive statistics for the overall cohort

Descriptive statistics for the overall cohort can be viewed in Tables 1, 2. Descriptive statistics for the GCS = 15 cohort can be viewed in Supplementary Tables 1, 2. The total cohort who met inclusion/exclusion criteria was 1,916 participants; of those, 63% followed up by 1 year post-injury. By 2 weeks post-injury, 43% of participants had followed up with a clinical provider, 4% had received inpatient rehabilitation, and 3% had received outpatient rehabilitation. By 3 months post-injury, 48% of participants had followed up with a clinical provider, 4% had received inpatient rehabilitation, and 10% had received outpatient rehabilitation. At 6 months post-injury, 28% indicated they had followed up with a clinical provider, 1% indicated they had received inpatient rehabilitation, and 7% indicated they had received outpatient rehabilitation in the last 3 months. At 12 months post-injury, 20% indicated they had followed up with a clinical provider, <1% indicated they had received inpatient rehabilitation and 5% indicated they had received outpatient rehabilitation in the last 3 months. Regardless of the type of clinical care, 84.5–88.3% of participants who followed up with a clinical provider within the first year reported that it had helped their recovery.

**Table 1:** Descriptive statistics for participants with presenting Glasgow Coma Scale score between 13 and 15 who received clinical follow-up care within the first year post-injury or was interested in clinical follow-up care in the first year post-injury*.

	Total Column %'s	Ever received clinical follow-up care Row %'s			Ever interested in clinical follow-up care Row %'s		
		No	Yes	Unk	No	Yes	Unk
Subjects	1916	618 (37%)	1,060 (63%)	238	886 (59%)	621 (41%)	409
Age							
Mean (SD)	41.8 (17.5)	37.9 (16.6)	43.4 (17.6)	44.5	39.7 (17.4)	41.7 (16.3)	46.4
Sex							
Male	1,269 (66%)	445 (40%)	656 (60%)	168	618 (61%)	388 (39%)	263
Female	647 (34%)	173 (30%)	404 (70%)	70	268 (53%)	233 (47%)	146
Race							
A - White	1,464 (77%)	461 (36%)	826 (64%)	177	703 (61%)	454 (39%)	307
B - Black	319 (17%)	116 (41%)	166 (59%)	37	122 (47%)	135 (53%)	62
C - Other	110 (6%)	36 (36%)	64 (64%)	10	57 (67%)	28 (33%)	25
Unknown	23	5 (56%)	4 (44%)	14	4 (50%)	4 (50%)	15
Hispanic							
No	1,507 (80%)	451 (34%)	882 (66%)	174	733 (62%)	458 (38%)	316
Yes	388 (20%)	162 (48%)	176 (52%)	50	148 (48%)	162 (52%)	78
Unknown	21	5 (71%)	2 (29%)	14	5 (83%)	1 (17%)	15
Education years							
Mean (SD)	13.5 (2.9)	13.1 (3.0)	13.9 (2.9)	12.8	13.8 (2.9)	13.1 (2.9)	13.3
Unknown	93	18	21	54	19	15	59
Insurance							
A - Private	1,180 (65%)	346 (32%)	732 (68%)	102	614 (64%)	345 (36%)	221
B - Medicaid	207 (11%)	68 (39%)	107 (61%)	32	84 (55%)	68 (45%)	55
C - Self pay	377 (21%)	169 (51%)	160 (49%)	48	150 (49%)	157 (51%)	70
D - Other	55 (3%)	16 (31%)	36 (69%)	3	21 (43%)	28 (57%)	6
Unknown	97	19 (43%)	25 (57%)	53	17 (43%)	23 (58%)	57
Patient type							
1 - ED only	509 (27%)	193 (43%)	260 (57%)	56	282 (68%)	132 (32%)	95
2 - Hospital admit	843 (44%)	285 (38%)	463 (62%)	95	408 (59%)	283 (41%)	152
3 - ICU admit	564 (29%)	140 (29%)	337 (71%)	87	196 (49%)	206 (51%)	162
ER Arrival GCS							
13	78 (4%)	16 (23%)	53 (77%)	9	30 (53%)	27 (47%)	21
14	361 (19%)	98 (31%)	218 (69%)	45	174 (62%)	105 (38%)	82
15	1,477 (77%)	504 (39%)	789 (61%)	184	682 (58%)	489 (42%)	306
LOC							
No	252 (14%)	84 (39%)	132 (61%)	36	121 (68%)	57 (32%)	74
Yes	1,576 (86%)	516 (37%)	872 (63%)	188	725 (57%)	542 (43%)	309
Unknown	88	18 (24%)	56 (76%)	14	40 (65%)	22 (35%)	26
PTA							
No	309 (18%)	113 (42%)	155 (58%)	41	156 (64%)	89 (36%)	64
Yes	1,436 (82%)	446 (35%)	814 (65%)	176	653 (57%)	484 (43%)	299
Unknown	171	59 (39%)	91 (61%)	21	77 (62%)	48 (38%)	46
Initial CT							
Negative	1,197 (64%)	461 (43%)	599 (57%)	137	607 (62%)	376 (38%)	214
Positive	660 (36%)	136 (24%)	435 (76%)	89	262 (54%)	220 (46%)	178
Unknown	59	21 (45%)	26 (55%)	12	17 (40%)	25 (60%)	17
TBI history							
None	1,371 (78%)	442 (36%)	784 (64%)	145	660 (60%)	446 (40%)	265
ED only	236 (13%)	86 (40%)	128 (60%)	22	107 (57%)	82 (43%)	47
Hospital admit	148 (8%)	50 (37%)	85 (63%)	13	60 (50%)	60 (50%)	28
Unknown	161	40 (39%)	63 (61%)	58	59 (64%)	33 (36%)	69
Psychiatric history							
No	1,492 (78%)	499 (38%)	799 (62%)	194	701 (59%)	482 (41%)	309
Yes	423 (22%)	119 (31%)	260 (69%)	44	184 (57%)	139 (43%)	100
Unknown	1	0 (0%)	1 (100%)	0	1 (100%)	0 (0%)	0
Migraine history							
No	1807 (94%)	591 (38%)	983 (62%)	233	836 (59%)	584 (41%)	387
Yes	108 (6%)	27 (26%)	76 (74%)	5	49 (57%)	37 (43%)	22
Unknown	1	0 (0%)	1 (100%)	0	1 (100%)	0 (0%)	0
Litigation by 12 m							
No	978 (79%)	321 (33%)	657 (67%)	0	556 (62%)	334 (38%)	88
Yes/Intends	265 (21%)	67 (25%)	198 (75%)	0	112 (48%)	122 (52%)	31
Unknown	673	230 (53%)	205 (47%)	238	218 (57%)	165 (43%)	290
Educ. materials							
No	654 (42%)	294 (45%)	360 (55%)	0	305 (51%)	290 (49%)	59
Yes	895 (58%)	276 (31%)	619 (69%)	0	509 (63%)	302 (37%)	84
Unknown	367	48 (37%)	81 (63%)	238	72 (71%)	29 (29%)	266
Contact info							
No	432 (27%)	217 (50%)	215 (50%)	0	188 (50%)	190 (50%)	54
Yes	1,150 (73%)	358 (31%)	792 (69%)	0	649 (62%)	404 (38%)	97
Unknown	334	43 (45%)	53 (55%)	238	49 (64%)	27 (36%)	258
Hospital call							
No	1,034 (65%)	429 (41%)	605 (59%)	0	565 (60%)	376 (40%)	93
Yes	563 (35%)	157 (28%)	406 (72%)	0	280 (56%)	224 (44%)	59
Unknown	319	32 (40%)	49 (60%)	238	41 (66%)	21 (34%)	257
Biomarkers Median values							
Day 1 GFAP	277	172	313	1895	253	280	377
Day 1 UCHL1	169	169	165	189	160	170	197

ED, emergency department; ER, emergency room; GCS, Glasgow Coma Scale; LOC, Loss of consciousness; PTA, Post-traumatic amnesia; CT, computed tomography; TBI, traumatic brain injury; Educ, educational; GFAP, Glial Fibrillary Acidic Protein; UCH-L1, Ubiquitin c-Terminal Hydrolase-L1.

**Table 2:** Participant responses regarding clinical follow-up clinical at 2 weeks, 3-, 6- and 12-months post-traumatic brain injury (TBI) with presenting Glasgow Coma Scale between 13 and 15.

Interview questions	2 weeks Since injury	3 months Since injury	6 months Last 3 Months	12 months Last 6 Months
Healthcare providers				
Have you seen any healthcare provider for your TBI?	43% (652/1510)	48% (681/1415)	28% (384/1348)	20% (255/1246)
Did it help?	87.4% (491/562)	88.3% (717/812)	86.5% (422/488)	84.5% (294/348)
Inpatient rehab				
Were you treated as an inpatient for problems related to your TBI?	4% (62/1538)	4% (60/1438)	1% (9/1358)	0% (2/1251)
Outpatient rehab				
Were you treated as an outpatient for problems related to your TBI?	3% (40/1539)	10% (145/1438)	7% (92/1358)	5% (59/1251)
Interested in follow-up care				
Yes, but…	38% (491/1291)	-	-	-
no/insufficient insurance coverage	12% (61/491)	-	-	-
insurance coverage was denied	1% (6/491)	-	-	-
could not arrange transportation	1% (5/491)	-	-	-
worried about the physical, emotional, or personal consequences	2% (11/491)	-	-	-
worried about the burden it would place on other close to me	2% (12/491)	-	-	-
treatment services have not yet been arranged	45% (221/491)	-	-	-
not given any information/referral	29% (144/491)	-	-	-
other	16% (81/491)	-	-	-
No, because…	62% (800/1291)	-	-	-
I did not think I needed it	92% (730/796)	-	-	-
I believe I can manage the problems caused by my injury on my own	9% (72/796)	-	-	-
I was dissatisfied with the treatment I received at the hospital	1% (6/796)	-	-	-

Only 38% of the overall sample reported they were interested in receiving follow-up care at the 2 week research visit. Of those participants, the most common reason for not following up with a clinical provider was “treatment services have not been arranged” (45%), “I was not given any information/referral (29%), “other- not specified” (16%), or “insufficient insurance coverage” (12%). Sixty-two percent of the sample indicated they were not interested in receiving clinical follow-up care by 2 weeks. Of those participants, the provided reasons for not being interested included “I did not think I needed it” (92%), “I believe I can manage the problems caused by my injury on my own” (9%), and “I was dissatisfied with the treatment I received at the hospital” (1%).

### Clinical follow-up rates stratified by interest in clinical follow-up

Follow-up rates and endorsement of whether follow-up helped their current condition or not can be found in Table 3. Overall, 61% of participants interested in clinical follow-up care reported receiving clinical follow-up care. Participants who received follow-up care reported that it helped at an 86% rate. Of those not interested in follow-up care, 42% reported receiving clinical follow-up care and 86% of those receiving care reported that it helped.

**Table 3:** Rates of clinical follow-up at 2 weeks and 3-months post-injury stratified by endorsing interest in receiving clinical follow-up care after traumatic brain injury with Glasgow Coma Scale 13 to 15 at presentation.

	2wk	3mo
Interested in clinical follow-up	491	124
Received clinical follow-up	299 (61%)	63 (52%)
Answered “no” to “did it help?”	22 (14%)	8 (17%)
Answered “yes” to “did it help?”	135 (86%)	39 (83%)
Unknown	32	16
Did not receive clinical follow-up	189 (39%)	59 (48%)
Unknown	3	2
Not interested in clinical follow-up	800	446
Received clinical follow-up	329 (42%)	198 (45%)
Answered “no” to “did it help?”	34 (14%)	11 (8%)
Answered “yes” to “did it help?”	208 (86%)	125 (92%)
Unknown	87	62
Did not receive clinical follow-up	458 (58%)	244 (55%)
Unknown	13	4

### Clinical outcomes for participants not interested in clinical follow-up

Clinical outcomes at 3-, 6-, and 12-months post-injury for participants who believed they did not need clinical follow-up at 2 weeks post-injury can be viewed in Table 4. Approximately 44% of participants who reported “I did not think I need follow-up” at 2-weeks (n = 730) had incomplete recovery (GOSE<8) at 12-months post-injury. Forty percent of participants who reported “I did not think I needed follow-up” had persistent symptoms at 12-months post-injury. Nineteen percent of participants who reported “I did not think I needed follow-up” had lower quality of life at 12-months post-injury. Approximately 39% of participants who reported “I can manage problems on my own” at 2 weeks post-injury (n = 72) had incomplete recovery (GOSE<8) at 12-months post-injury. Thirty one percent of participants who reported “I can manage problems on my own” had persistent symptoms at 12-months post-injury. Fourteen percent of participants who reported “I can manage problems on my own” had lower quality of life at 12-months post-injury.

**Table 4:** Rates of poor long-term outcomes by 3-, 6-, and 12-months in participants with presenting Glasgow Coma Scale between 13 and 15 who were not interested in clinical follow-up at 2 weeks post-injury.

	3 Months			6 Months			12 Months		
	GOSE<8	RPQ 3+	QOL ≤ 51	GOSE<8	RPQ 3+	QOL ≤ 51	GOSE<8	RPQ 3+	QOL ≤ 51
I did not think I needed clinical follow-up									
Agree (*n* = 730)	55.8% (333/597)	44.6% (281/630)	18.6% (117/629)	51.0% (296/580)	39.6% (241/608)	19.5% (118/606)	43.7% (243/556)	40.1% (232/578)	19.4% (112/577)
Disagree (*n* = 60)	64% (37/58)	53% (32/60)	23% (14/60)	48% (24/50)	50% (26/52)	15% (8/52)	23% (15/45)	45% (21/47)	15% (7/47)
I believe I can manage problems on my own									
Yes (*n* = 72)	54.7% (35/64)	50% (33/66)	24.2% (16/66)	48.3% (28/58)	46.7 (28/60)	13.3% (8/60)	38.8% (19/49)	41.2% (21/51)	13.7% (7/51)
No (*n* = 624)	66% (331/591)	45% (280/624)	18% (115/623)	51% (292/572)	40% (249/600)	20% (118/598)	43% (239/552)	40% (232/574)	20% (112/573)

Glasgow Outcome Scale Extended (GOSE); Rivermead Post-Concussion Questionnaire (RPQ); Quality of Life After Brain Injury-Overall Scale (QOL); cutoffs for these outcomes were determined based upon prior research.

### Follow-up rates for clinically relevant subgroups

**Figure 1:**
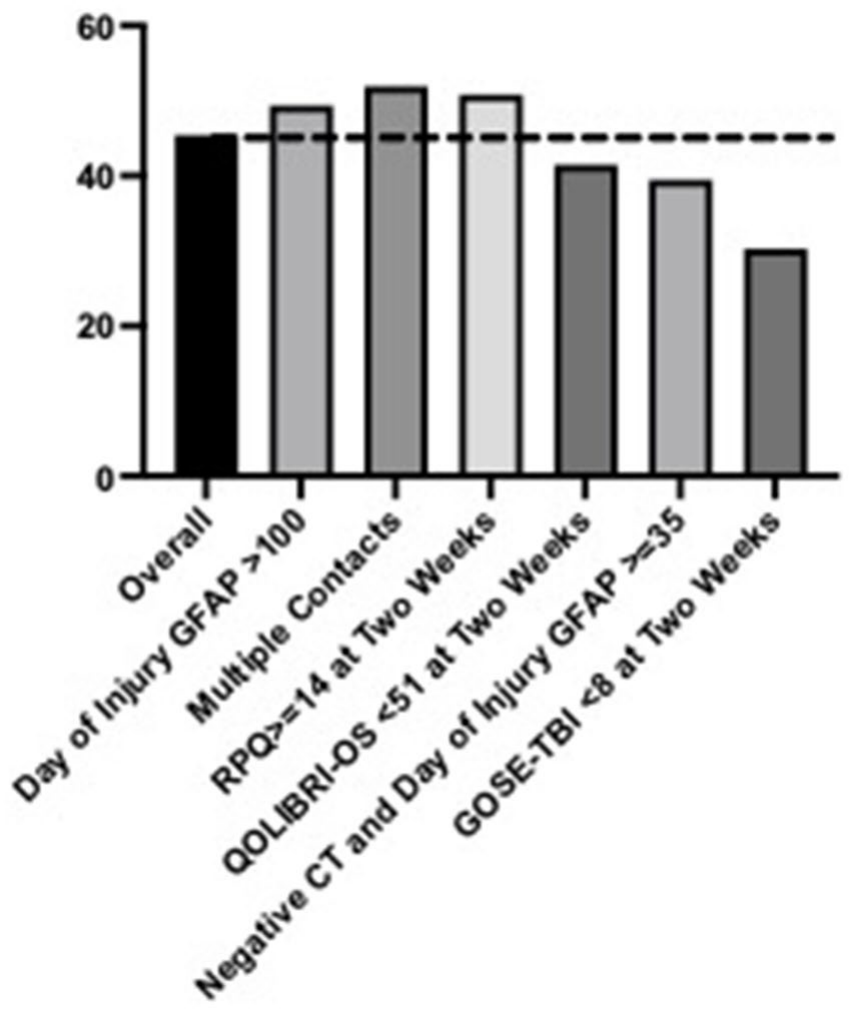
Clinical follow up rates (%) for the overall sample by 2 weeks post-injury with presenting Glasgow Coma Scale score between 13 and 15 and by high-risk subgroups.

**Figure 2:**
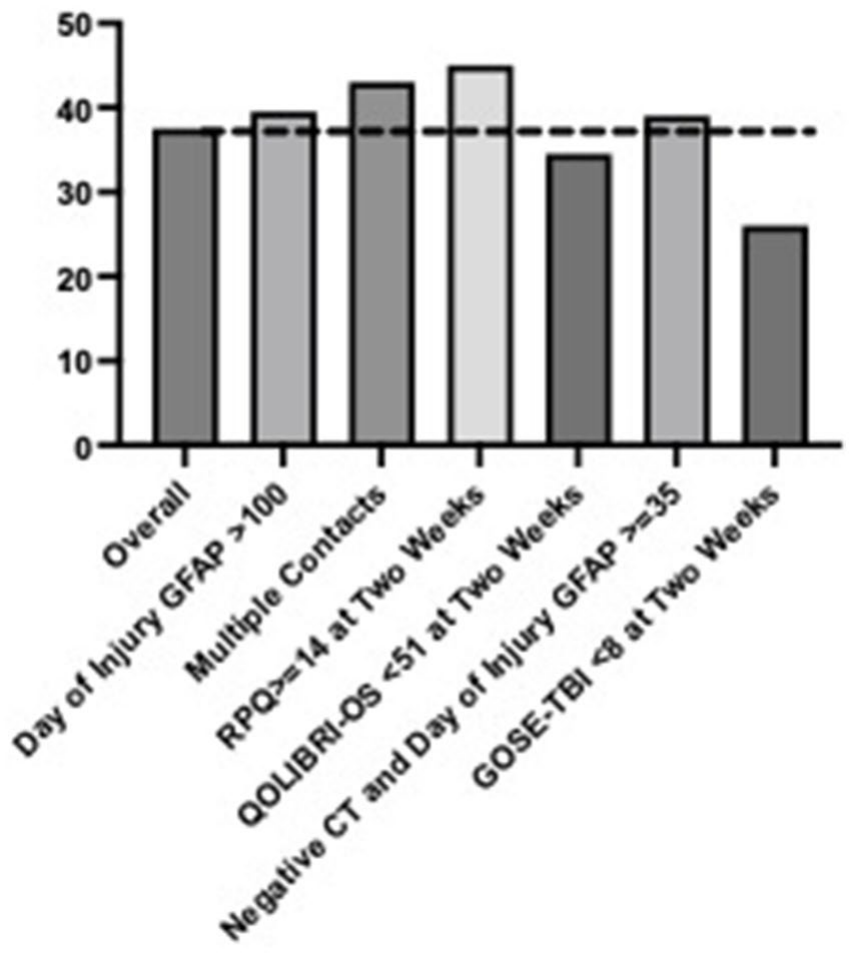
Clinical follow up rates (%) for the overall sample by 2 weeks post-injury with presenting Glasgow Coma Scale score of 15 and by high-risk subgroups.

Follow up rates for subgroups can be viewed in Figures 1, 2. For participants with GCS 13–15, 43% of participants followed up by 2 weeks and 48% followed up by 3 months. Participants with day of injury GFAP ≥100, multiple hospital contacts and a total RPQ score ≥ 14 had increased follow up rates compared to the overall cohort (2 weeks: 46–49%, 3 months: 53–55%). Participants with QOLIBRI-OS <51 at 2 weeks, GOSE TBI < 8 at 2 weeks, or negative head CT but day of injury GFAP≥35 had decreased rates of follow up compared to the overall cohort (2 weeks: 34–42%, 3 months: 27–41%). Similar trends were observed for participants with presenting GCS = 15 (see Supplementary Table 3).

## Discussion

In this secondary analysis of a prospective cohort study with 1,916 participants with TBI and presenting GCS score between 13 and 15, less than half of participants followed up with a clinical provider by 3 months post-injury and 37% of participants had not followed up by 1 year post-injury. Clinical markers of injury severity (i.e., lower GCS, positive head CT scan, admission to the ICU), prior medical history (i.e., psychiatric disorder, migraines), and hospital outreach increased rates of clinical follow up. Nearly 2 in 3 participants indicated they were not interested in receiving clinical follow-up care at 2 weeks post-injury. Participants not interested in follow-up care had high rates of poor functional recovery (44%), persistent symptoms (40%) and lower quality of life after brain injury (19%) at 12 months post-injury. Education and provider emphasis on the importance of clinical follow-up after hospital discharge with TBI need to be enhanced. Acute evaluation of blood biomarkers may represent a more sensitive, objective assessment to facilitate these processes.

Based upon the results of this study, it is likely that provider emphasis on the need for follow up care was more pronounced for participants with traditional, objective markers of TBI severity (e.g., lower GCS, positive head CT scan, admission to the ICU). This is evidenced by the finding that participants with a negative head CT scan but elevated GFAP (≥35 pg./mL) followed up at a 39% rate while participants with a positive head CT followed up at 76% (Figure 2). Prior work assessing the TRACK-TBI cohort reported the median GFAP level for patients with orthopedic injuries and healthy controls to be 13.1 and 8 pg./mL, respectively (13). In an otherwise healthy adult being evaluated for potential TBI with negative head CT scan, day-of-injury GFAP may be useful for aiding in diagnosis. For the present study, blood biomarker results were not available for provider use and blood samples were collected for research purposes. This is the first known study to report clinical follow-up after TBI with GCS 13 to 15 in adults presenting to a level 1 trauma center ED in relation to acute blood biomarkers, but future studies will be necessary to understand how clinical use of these blood biomarkers impacts clinical follow-up rates.

The TBI patient’s long-term recovery may be enhanced by targeted assessment at 2 weeks post-injury. A meaningful percentage of participants with RPQ total scores≥14, QOLIBRI-OS<51, and incomplete functional recovery (i.e., GOSE-TBI < 8) at 2-weeks had not followed up by 3 months post-injury (27–55%). This finding is notable as TBI patients who meet these criteria at 2 weeks have increased odds of worse long-term outcomes from their injury. The RPQ and QOLIBRI-OS are brief symptom surveys which could be completed over the phone. Doing so at approximately 2 weeks post-injury has the potential to enhance patient education and subsequent clinical follow up, as participants who reported receiving educational materials and/or a phone call from the hospital had higher follow-up rates than those who did not (Table 1).

There is robust evidence to support the benefits of early follow up care (i.e., within the first week of injury) for “mild” TBI in other populations (19, 20), but little to no evidence for early follow up care in adult populations first evaluated at a level 1 trauma center. Improving both patient and provider education on this topic and identifying barriers to better follow-up implementation practices are critical areas of future research (10, 21). Increasing advocacy for TBI as a potentially chronic condition and public health problem could increase awareness from patients and stakeholders (11, 22). Based upon results from other populations, such as those with sport-related concussion, earlier initiation of active rehabilitation is likely to reduce risk for long-term sequelae (23, 24). There are many population-based challenges between patients who suffer a sport-related concussion compared to patients evaluated at the ED for a “mild” TBI, including higher rates of public insurance/self-pay, availability of follow-up treatment pathways, and differing social determinants of health profiles (11, 25). These multifactorial challenges need to be addressed in the development of an effective system for follow-up care for this population.

### Limitations

This study has limitations. Analyzing follow up clinical provider type (i.e., primary care physician, physical therapist, specialty clinic, etc.) was not possible due to large amounts of missing data. Understanding the impact of specific types of care will further improve patient triage and education efforts. Demographics, medical history and hospital provision of education materials, contact information and whether a follow-up call from the hospital was conducted were self-reported and may have been subject to recall bias.

## Conclusion

Prioritizing timely clinical follow-up for adult patients with TBI with GCS 13 to 15 is critical for improving rates of long-term recovery in this population. The results of this study suggest that clinical follow-up is not common by 2 weeks post-injury and, perhaps more importantly, most patients do not have interest or see the need for follow-up care. Follow-up was relatively low even among patients experiencing persistent symptoms after mTBI (mild Traumatic Brain Injury). Patient and ED provider education on the importance of clinical follow-up is necessary. Patients who received more information/outreach from the hospital were more likely to follow-up. The primary reasons for lack of interest in follow-up care were based on the patient’s impression that it was not necessary or that connections to follow-up care had not been facilitated for them. Incorporation of blood biomarkers in ED clinical assessment requires future study, as patient education regarding TBI prognosis with a negative head CT scan may improve follow up rates, as well.

## Group member of The TRACK-TBI Investigators

C. Dirk Keene, University of Washington; Frederick K. Korley, University of Michigan; Vijay Krishnamoorthy, Duke University; Christine Mac Donald, University Washington; Randall Merchant, Virginia Commonwealth University; Pratik Mukherjee, University of California, San Francisco; Laura B. Ngwenya, University of Cincinnati; Ava Puccio, University of Pittsburgh; Claudia Robertson, Baylor College of Medicine; Richard B Rodgers, Goodman Campbell Brain and Spine; Sabrina R. Taylor, University of California, San Francisco; Ross Zafonte, Harvard Medical School.

## Data Availability

The datasets presented in this study can be found in online repositories. The names of the repository/repositories and accession number(s) can be found below: https://fitbir.nih.gov./study_profile/267.
