# Supplementary material for: Follow up rates and patient interest in clinical care after mild traumatic brain injury presenting to a level 1 trauma center: a TRACK-TBI prospective cohort study
Source: Front Neurol. 2025 Apr 2;16:1558204. doi: 10.3389/fneur.2025.1558204 (PMC12002085; doi:10.3389/fneur.2025.1558204)
Supplement: Supplementary file 1 [file Table_1.docx]

| **Supplemental Table 1. Descriptive statistics for participants with presenting Glasgow Coma Scale score of 15 who received clinical follow-up within the first year post-injury or was interested in clinical follow-up in the first year post-injury** | | | | | | | | |
| --- | --- | --- | --- | --- | --- | --- | --- | --- |
|  | **Total** Column %'s | **Ever Received Clinical Follow-Up**  Row %'s | | | **Ever Interested in Clinical Follow-Up**  Row %'s | | |  |
|  |  | No | Yes | Unk | No | Yes | Unk |  |
| **Subjects** | **972** | **382** (45%) | **475** (55%) | **115** | **484** (61%) | **310** (39%) | **178** |  |
| **Age** |  |  |  |  |  |  |  |  |
| Mean (SD) | 38.4 (15.8) | 35.2 (15.2) | 40.9 (15.8) | 39.0 | 37.8 (16.3) | 38.5 (14.8) | 40.0 |  |
| **Sex** |  |  |  |  |  |  |  |  |
| Male | 619 (64%) | 268 (50%) | 269 (50%) | 82 | 323 (64%) | 179 (36%) | 117 |  |
| Female | 353 (36%) | 114 (36%) | 206 (64%) | 33 | 161 (55%) | 131 (45%) | 61 |  |
| **Race** |  |  |  |  |  |  |  |  |
| A - White | 698 (73%) | 276 (45%) | 342 (55%) | 80 | 360 (63%) | 216 (38%) | 122 |  |
| B - Black | 208 (22%) | 80 (44%) | 101 (56%) | 27 | 89 (53%) | 79 (47%) | 40 |  |
| C - Other | 56 (6%) | 22 (42%) | 30 (58%) | 4 | 32 (73%) | 12 (27%) | 12 |  |
| Unknown | 10 | 4 (67%) | 2 (33%) | 4 | 3 (50%) | 3 (50%) | 4 |  |
| **Hispanic** |  |  |  |  |  |  |  |  |
| No | 782 (81%) | 290 (42%) | 404 (58%) | 88 | 406 (63%) | 240 (37%) | 136 |  |
| Yes | 181 (19%) | 88 (56%) | 70 (44%) | 23 | 74 (52%) | 69 (48%) | 38 |  |
| Unknown | 9 | 4 (80%) | 1 (20%) | 4 | 4 (80%) | 1 (20%) | 4 |  |
| **Education Years** |  |  |  |  |  |  |  |  |
| Mean (SD) | 13.4 (2.7) | 13.0 (2.7) | 13.9 (2.7) | 12.3 | 13.8 (2.8) | 13.0 (2.6) | 12.7 |  |
| Unknown | 35 | 8 | 5 | 22 | 8 | 4 | 23 |  |
| **Insurance** |  |  |  |  |  |  |  |  |
| A - Private | 593 (64%) | 226 (41%) | 324 (59%) | 43 | 336 (66%) | 176 (34%) | 81 |  |
| B - Medicaid | 119 (13%) | 43 (43%) | 58 (57%) | 18 | 46 (53%) | 41 (47%) | 32 |  |
| C - Self Pay | 195 (21%) | 97 (58%) | 70 (42%) | 28 | 85 (54%) | 72 (46%) | 38 |  |
| D - Other | 25 (3%) | 8 (35%) | 15 (65%) | 2 | 9 (41%) | 13 (59%) | 3 |  |
| Unknown | 40 | 8 (50%) | 8 (50%) | 24 | 8 (50%) | 8 (50%) | 24 |  |
| **Patient Type** |  |  |  |  |  |  |  |  |
| 1 - ED Only | 377 (39%) | 151 (45%) | 188 (55%) | 38 | 204 (66%) | 103 (34%) | 70 |  |
| 2 - Hospital Admit | 458 (47%) | 183 (46%) | 218 (54%) | 57 | 225 (59%) | 157 (41%) | 76 |  |
| 3 - ICU Admit | 137 (14%) | 48 (41%) | 69 (59%) | 20 | 55 (52%) | 50 (48%) | 32 |  |
| **ER Arrival GCS** |  |  |  |  |  |  |  |  |
| 15 | 972 (100%) | 382 (45%) | 475 (55%) | 115 | 484 (61%) | 310 (39%) | 178 |  |
| **LOC** |  |  |  |  |  |  |  |  |
| No | 132 (14%) | 53 (46%) | 62 (54%) | 17 | 70 (71%) | 29 (29%) | 33 |  |
| Yes | 806 (86%) | 322 (45%) | 393 (55%) | 91 | 399 (59%) | 274 (41%) | 133 |  |
| Unknown | 34 | 7 (26%) | 20 (74%) | 7 | 15 (68%) | 7 (32%) | 12 |  |
| **PTA** |  |  |  |  |  |  |  |  |
| No | 210 (24%) | 94 (50%) | 94 (50%) | 22 | 117 (67%) | 58 (33%) | 35 |  |
| Yes | 667 (76%) | 247 (43%) | 333 (57%) | 87 | 320 (59%) | 221 (41%) | 126 |  |
| Unknown | 95 | 41 (46%) | 48 (54%) | 6 | 47 (60%) | 31 (40%) | 17 |  |
| **Initial CT** |  |  |  |  |  |  |  |  |
| Negative | 972 (100%) | 382 (45%) | 475 (55%) | 115 | 484 (61%) | 310 (39%) | 178 |  |
| **TBI History** |  |  |  |  |  |  |  |  |
| None | 673 (75%) | 270 (44%) | 338 (56%) | 65 | 355 (63%) | 212 (37%) | 106 |  |
| ED Only | 133 (15%) | 55 (46%) | 65 (54%) | 13 | 59 (55%) | 48 (45%) | 26 |  |
| Hospital Admit | 88 (10%) | 37 (47%) | 42 (53%) | 9 | 35 (49%) | 37 (51%) | 16 |  |
| Unknown | 78 | 20 (40%) | 30 (60%) | 28 | 35 (73%) | 13 (27%) | 30 |  |
| **Psych History** |  |  |  |  |  |  |  |  |
| No | 749 (77%) | 305 (47%) | 344 (53%) | 100 | 380 (62%) | 231 (38%) | 138 |  |
| Yes | 222 (23%) | 77 (37%) | 130 (63%) | 15 | 103 (57%) | 79 (43%) | 40 |  |
| Unknown | 1 | 0 (0%) | 1 (100%) | 0 | 1 (100%) | 0 (0%) | 0 |  |
| **Migraine History** |  |  |  |  |  |  |  |  |
| No | 899 (93%) | 366 (47%) | 420 (53%) | 113 | 451 (61%) | 283 (39%) | 165 |  |
| Yes | 72 (7%) | 16 (23%) | 54 (77%) | 2 | 32 (54%) | 27 (46%) | 13 |  |
| Unknown | 1 | 0 (0%) | 1 (100%) | 0 | 1 (100%) | 0 (0%) | 0 |  |
| **Litigation by 12m** |  |  |  |  |  |  |  |  |
| No | 471 (76%) | 187 (40%) | 284 (60%) | 0 | 284 (64%) | 158 (36%) | 29 |  |
| Yes/Intends | 151 (24%) | 49 (32%) | 102 (68%) | 0 | 72 (52%) | 67 (48%) | 12 |  |
| Unknown | 350 | 146 (62%) | 89 (38%) | 115 | 128 (60%) | 85 (40%) | 137 |  |
| **Educ. Materials** |  |  |  |  |  |  |  |  |
| No | 347 (43%) | 181 (52%) | 166 (48%) | 0 | 183 (57%) | 139 (43%) | 25 |  |
| Yes | 451 (57%) | 177 (39%) | 274 (61%) | 0 | 266 (63%) | 153 (37%) | 32 |  |
| Unknown | 174 | 24 (41%) | 35 (59%) | 115 | 35 (66%) | 18 (34%) | 121 |  |
| **Contact Info** |  |  |  |  |  |  |  |  |
| No | 239 (29%) | 140 (59%) | 99 (41%) | 0 | 123 (56%) | 95 (44%) | 21 |  |
| Yes | 574 (71%) | 222 (39%) | 352 (61%) | 0 | 340 (63%) | 200 (37%) | 34 |  |
| Unknown | 159 | 20 (45%) | 24 (55%) | 115 | 21 (58%) | 15 (42%) | 123 |  |
| **Hospital Call** |  |  |  |  |  |  |  |  |
| No | 544 (67%) | 270 (50%) | 274 (50%) | 0 | 318 (63%) | 189 (37%) | 37 |  |
| Yes | 273 (33%) | 94 (34%) | 179 (66%) | 0 | 145 (57%) | 108 (43%) | 20 |  |
| Unknown | 155 | 18 (45%) | 22 (55%) | 115 | 21 (62%) | 13 (38%) | 121 |  |
| **Biomarkers** Median Values |  |  |  |  |  |  |  |  |
| Day 1 GFAP |  | 87.9 | 122 | --- | 96.9 | 119.6 | 73.5 |  |
| Day 1 UCHL1 |  | 156 | 143 | --- | 142 | 150 | 164 |  |

Key: ED= emergency department, ER= emergency room, GCS= Glasgow Coma Scale, LOC= Loss of consciousness, PTA= Post-traumatic amnesia, CT= computed tomography, TBI= traumatic brain injury, Educ= educational, GFAP= Glial Fibrillary Acidic Protein, UCH-L1= Ubiquitin c-Terminal Hydrolase-L1
