# Supplementary material for: Follow up rates and patient interest in clinical care after mild traumatic brain injury presenting to a level 1 trauma center: a TRACK-TBI prospective cohort study
Source: Front Neurol. 2025 Apr 2;16:1558204. doi: 10.3389/fneur.2025.1558204 (PMC12002085; doi:10.3389/fneur.2025.1558204)
Supplement: Supplementary file 2 [file Table_2.docx]

| **Supplemental Table 2. Responses regarding clinical follow-up at 2 weeks, 3-, 6- and 12-months following traumatic brain injury with presenting Glasgow Coma Scale of 15 and negative head computed tomography scan on hospital arrival** | | | | |
| --- | --- | --- | --- | --- |
| **Interview Questions** | **2 Weeks** Since Injury | **3 Months** Since Injury | **6 Months**  Last 3 Months | **12 Months**  Last 6 Months |
| **Healthcare Providers** |  |  |  |  |
| Have you seen any healthcare provider for your TBI? | 37% (290/775) | 38% (272/725) | 22% (155/694) | 17% (109/625) |
| Did it help? | 86.8% (190/219) | 88.1% (260/295) | 88.1% (163/185) | 79.7% (110/138) |
| **Inpatient Rehab** |  |  |  |  |
| Were you treated as an inpatient for problems related to your TBI? | 2% (19/796) | 2% (16/741) | 0% (2/697) | 0% (1/627) |
| **Outpatient Rehab** |  |  |  |  |
| Were you treated as an outpatient for problems related to your TBI? | 1% (10/796) | 7% (53/741) | 5% (32/697) | 4% (24/627) |
| **Interested in Follow-Up Care** |  |  |  |  |
| Yes, but... | 36% (248/688) | --- | --- | --- |
| no/insufficient insurance coverage | 15% (36/248) | --- | --- | --- |
| insurance coverage was denied | 1% (3/248) | --- | --- | --- |
| could not arrange transportation | 1% (3/248) | --- | --- | --- |
| worried about the physical, emotional, or personal consequences | 4% (9/248) | --- | --- | --- |
| worried about the burden it would place on other close to me | 4% (9/248) | --- | --- | --- |
| treatment services have not yet been arranged | 36% (90/248) | --- | --- | --- |
| not given any information/referral | 31% (78/248) | --- | --- | --- |
| other | 21% (51/248) | --- | --- | --- |
| No, because... | 64% (440/688) | --- | --- | --- |
| I did not think I needed it | 93% (408/438) | --- | --- | --- |
| I believe I can manage the problems caused by my injury on my own | 8% (33/438) | --- | --- | --- |
| I was dissatisfied with the treatment I received at the hospital | 1% (4/438) | --- | --- | --- |
