# Supplementary material for: Follow up rates and patient interest in clinical care after mild traumatic brain injury presenting to a level 1 trauma center: a TRACK-TBI prospective cohort study
Source: Front Neurol. 2025 Apr 2;16:1558204. doi: 10.3389/fneur.2025.1558204 (PMC12002085; doi:10.3389/fneur.2025.1558204)
Supplement: Supplementary file 3 [file Table_3.docx]

| **Supplemental Table 3. Rates of clinical follow-up by 2 weeks and 3 months post-injury by clinically relevant subgroups*** | | | | | | |
| --- | --- | --- | --- | --- | --- | --- |
|  | **N** | **GCS=13-15 Clinical Follow-Up** | | **N** | **GCS=15 Clinical Follow-Up** | |
|  |  | **2 Weeks** | **3 Months** |  | **2 Weeks** | **3 Months** |
| **Full Sample** | 1916 | 652/1510  43% | 681/1415  48% | 972 | 290/775  37% | 272/725  38% |
| **Day 1 GFAP ≥100** | 1247 | 467/1009  46% | 496/943  53% | 465 | 152/382  40% | 141/358  39% |
| **GOSE TBI <8 at 2 weeks** | 166 | 54/160  34% | 38/141  27% | 115 | 30/110  27% | 25/100  25% |
| **RPQ ≥14 at 2 weeks** | 832 | 372/795  47% | 393/710  55% | 415 | 171/396  43% | 164/349  47% |
| **QOLIBRI-OS<51 at 2 weeks** | 830 | 342/811  42% | 290/705  41% | 443 | 160/430  37% | 124/385  32% |
| **Multiple Contacts** | 938 | 414/852  49% | 435/790  55% | 467 | 175/422  41% | 180/398  45% |
| **Negative CT & Day 1 GFAP≥35** | 823 | 260/672  39% | 252/628  40% | 638 | 203/519  39% | 189/486  39% |

*Key: CT= Computed tomography, GCS= Glasgow Coma Scale, GFAP= Glial Fibrillary Acidic Protein, GOSE= Glasgow Outcome Scale Extended, RPQ= Rivermead post-Concussion Questionnaire, QOLIBRI-OS= Quality of Life after Brain Injury Overall Scale
